# Factors Affecting Hypertension among the Malaysian Elderly

**DOI:** 10.3390/jcdd3010008

**Published:** 2016-03-02

**Authors:** Sima Ataollahi Eshkoor, Tengku Aizan Hamid, Suzana Shahar, Chee Kyun Ng, Chan Yoke Mun

**Affiliations:** 1 Malaysian Research on Aging (MyAging), University Putra Malaysia, Serdang 43400, Malaysia; simaataolahi@yahoo.com (S.A.E.); mpnck@eng.upm.edu.my (C.K.N.); cym@upm.edu.my (C.Y.M.); 2 Iranian Research Center on Aging, University of Social Welfare and Rehabilitation Sciences, Tehran 1985713834, Iran; 3 Faculty of Health Sciences, Universiti Kebangsaan Malaysia, Kuala Lumpur 50300, Malaysia; suzanas@medic.ukm.my

**Keywords:** age, albumin, body weight, cholesterol, glucose, hypertension, triglyceride

## Abstract

Hypertension is a common chronic disease in the elderly. This study aimed to determine the effects of age, ethnicity, gender, education, marital status, nutritional parameters, and blood elements on the risk of high blood pressure in the Malaysian elderly. This research was conducted on a group of 2322 non-institutionalized Malaysian elderly. The hierarchy binary logistic regression analysis was applied to estimate the risk of hypertension in respondents. Approximately, 45.61% of subjects had hypertension. The findings indicated that the female gender (Odds ratio (OR) = 1.54), an increase in body weight (OR = 1.61), and an increase in the blood levels of albumin (OR = 1.51), glucose (OR = 1.92), and triglycerides (OR = 1.27) significantly increased the risk of hypertension in subjects (*p* < 0.05). Conversely, an increase in both dietary carbohydrates (OR = 0.74), and blood cholesterol level (OR = 0.42) significantly reduced the risk of hypertension in samples (*p* < 0.05). Furthermore, the results showed that ethnicity was a non-relevant factor to increase the risk of hypertension in subjects. It was concluded that female gender, an increase in body weight, and an increase in the blood levels of glucose, triglycerides, and albumin enhanced the risk of high blood pressure in the Malaysian elderly. In addition, an increase in both dietary carbohydrates and blood cholesterol level decreased hypertension in subjects.

## 1. Introduction

Hypertension is a common chronic condition in the elderly that is diagnosed with a consistent high blood pressure [1]—systolic pressure equal to or greater than 140 mm Hg and/or diastolic pressure equal to or greater than 90 mm Hg [2,3] for a long time [1]. It can cause and accelerate vascular complications via induced changes in the structure and function of endothelial cells [4]. Many factors such as age, obesity [3], alcohol intake, and social class [5] can affect the risk of hypertension.

Although hypertension is not a normal part of aging [5], its rate elevates markedly with age [2]. It affects almost two thirds of elderly over aged 60 years [2]; more than roughly 50% of those aged ≥60 years and approximately 66% of aged ≥65 years [6]. Hypertension also affects the Malaysian elderly. Its prevalence in the elderly in urban and rural areas is 62% and 26%, respectively [7]. Approximately 58.3% of elderly in northern Malaysia have hypertension. The prevalence of hypertension in central Malaysia is 25.6% and 51.1% in community and nursing homes, respectively, that is among people aged 55 years and above [8].

However, hypertension is an important cause of morbidity and mortality in the elderly [8,9]. It can lead to events including stroke, heart failure, coronary artery disease, and end-stage renal disease in the elderly population [9]. The underlying cause of hypertension in the elderly is the relative changes in the arterial resistance and stiffness [5] that is characterized by (1) the increased peripheral vascular resistance, (2) the age-related neurohormonal and autonomic dysregulation, and (3) the autoregulation dysfunction in major target-organs such as the brain, heart, and kidneys [10,11].

Lowering blood pressure can reduce the risk of mortality and morbidity in hypertensive patients [12]. Lifestyle modifications such as diet and exercise are the first treatments in hypertension. Accordingly, low sodium diet, weight control, higher physical activity, the limit of alcohol intake, and the adoption of the Dietary Approaches to Stop Hypertension (DASH) are the main factors to control blood pressure [6]. Diet plays an important role in the management of age-related hypertension [3]. Thus, dietary patterns, including vegetarian diets,Mediterranean-style diets, and diets constituting fruits, grains, nuts, vegetables, poultry, fish, unsaturated vegetable oils, low-fat dairy products, less sugar-containing beverages, less sweets, and the smaller amounts of red meat can decrease such blood pressure [3].

Despite the importance of lifestyle modifications on hypertension [13], however, there are some other factors such as blood elements that may have significant effects on hypertension and need to be studied. Since hypertension is a common problem among the Malaysian elderly [8], and the concurrent effects of sociodemographic factors, blood elements, and nutritional parameters on hypertension have not been yet investigated in the Malaysian elderly, this study aimed to determine the effects of the above factors on the risk of hypertension in those people. It may facilitate identifying the exact risks of hypertension in different populations.

## 2. Methodology

The main project with the code of NN-060-2013 was a heterogeneous survey entitled “Neuroprotective Model for Healthy Longevity among the Malaysian Elderly” carried out in co-operation with the Universiti Kebangsaan Malaysia (UKM), and the Malaysian Research Institute on Ageing (MyAgeing), Universiti Putra Malaysia (UPM). The approval and permission for conducting the study were received from the Ethical Committee of the Universiti Kebangsaan Malaysia (UKM).

The main project recruited 2332 elderly who were Malaysian aged 60 years and above living in non-institutional places. This study as a part of the main project involved 2322 subjects after considering missing data. Collected samples were of different ethnicities—Malays, Chinese, Indians and others. The elderly who were bedridden and living in institutions were excluded. Participants were gathered at community halls or centers for interviews and health screening. The written consent was taken from subjects prior to interview. The trained fieldworkers conducted a face-to-face interview.

This study evaluated the effects of variables including, socio-demographic factors, nutritional status, and body weight as well as the blood levels of glucose, albumin, triglycerides, and cholesterol on the risk of high blood pressure in respondents. Questionnaires were used to collect data about socio-demographic factors (age, gender, ethnicity, educational level, and marital status), hypertension and nutritional status. The data were classified into two subgroups based on the median of variables, which facilitated regression analysis. Age was dichotomized into (1) “less than 75 years” and (2) “75 years and above”. The report of hypertension was in accordance with physician approval and/or taking medications. Subjects were coded as no having hypertension (0) and having hypertension (1). The evaluation of nutritional dietary intake of compounds was done by History Questionnaires (DHQ) and Nutritionist Pro 3. Venous blood samples were taken for determining the effects of triglycerides, cholesterol, glucose, and albumin levels on the risk of hypertension in the Malaysian elderly.

### Statistical Analysis

A three-step hierarchical binary logistic regression model using SPSS version 22.0 (SPSS Inc., Chicago, IL, USA) was used to test the effects of demographic factors, body weight, nutritional status, and the blood levels of glucose, albumin, triglycerides, and cholesterol on the risk of hypertension in subjects. Prior to the hierarchical regression analyses, correlation tests were examined to determine the existence of associations between the dependent variable and covariates. The first step of analysis assessed the effects of demographic factors and body weight on the risk of high blood pressure. The second model was built on Model 1 by adding dietary properties including thiamin, carbohydrates and energy. In the third step, the increase in the blood levels of cholesterol, triglycerides, albumin, and glucose was evaluated. Odds ratios (OR) with 95% confidence intervals (95% CI) were computed. The critical level for rejection of null hypothesis was considered to be a *p* value of 5%, two-tailed.

## 3. Results

Various analyses were run on data collected from 2322 subjects who were the Malaysian elderly and non-institutionalized. The prevalence of hypertension in respondents was approximately 45.61% (95% CI: 43.59–47.64) (Table 1).

The percentage of hypertension in samples with regard to variables has been summarized in Table 2, Table 3 and Table 4. The results showed that the prevalence of hypertension in the age groups of (1) less than 75 years and (2) 75 years and above was 45.78% and 44.94%, respectively. The percentage of hypertension in females (48.18%) was found to be higher than males (42.82%). In addition, non-married subjects (48.44%) displayed a greater rate of hypertension compared to married ones (44.29%). The rate of hypertension in educated subjects (43.29%) was less than non-educated ones (46.23%). Among all samples, non-Malays (49.71%) had a higher rate of hypertension than Malays (43.12%).

The bivariate analysis established the association between hypertension and each of the variables by chi-square tests. A significant association was found between the risk of hypertension and each variable of gender (χ^2^ = 6.71, *p* = 0.010), ethnicity (χ^2^ = 9.55, *p* = 0.002), body weight (χ^2^ = 136.49, *p* < 0.001), and the blood levels of albumin (χ^2^ = 14.00, *p* < 0.001), cholesterol (χ^2^ = 55.04, *p* < 0.001), triglycerides (χ^2^ = 6.03, *p* = 0.014), and glucose (χ^2^ = 54.25, *p* < 0.001), as well as the dietary levels of energy (χ^2^ = 4.78, *p* = 0.029), carbohydrates (χ^2^ = 14.91, *p* < 0.001), and thiamin (χ^2^ = 5.08, *p* = 0.024) (Table 2, Table 3 and Table 4).

As this study aimed to test the effects of socio-demographic factors, blood elements, and nutritional components on the risk of hypertension, a three-step hierarchical regression analysis was used. The results showed that female gender (*p* < 0.001), an increase in body weight (*p* < 0.001), and an increase in the blood levels of glucose (*p* < 0.001), albumin (*p* < 0.001), and triglycerides (*p* = 0.025) significantly enhanced the risk of hypertension in respondents (*p* < 0.01). The increase of both blood cholesterol level (*p* < 0.001) and dietary carbohydrates (*p* = 0.044) significantly reduced the risk of hypertension in subjects. In addition, the effects of factors including ethnicity, carbohydrates to fat ratio, and the dietary level of both thiamin and energy on hypertension were not significant (*p* > 0.05). The results have been summarized in Table 5.

Model 1 showed the significant effects of gender (*p* = 0.001; OR = 1.43), ethnicity (*p* = 0.047; OR = 0.82), and body weight (*p* < 0.001; OR = 1.88) on the risk of hypertension in subjects. It was found that female gender and higher body weight increased the risk of hypertension, but Malay ethnicity reduced the risk. In Model 2, female gender (*p* = 0.007; OR = 1.34) and increase in body weight (*p* < 0.001; OR = 1.90) significantly enhanced the risk of hypertension in respondents, but a high-dietary carbohydrate reduced the risk (*p* = 0.026; OR = 0.72). The significant effects of gender (*p* < 0.001; OR = 1.54), dietary carbohydrate (*p* = 0.044; OR = 0.74), body weight (*p* < 0.001; OR = 1.61), as well as the blood levels of variables, including triglycerides (*p* = 0.025; OR = 1.27), glucose (*p* < 0.001; OR = 1.92), cholesterol (*p* < 0.001; OR = 0.42), and albumin (*p* < 0.001; OR = 1.51) were found in Model 3. The findings indicated that female gender and an increase in the factors of body weight and the blood levels of triglycerides, albumin, and glucose enhanced the risk of hypertension in subjects. The increased blood level of glucose was the most effective factor to elevate blood pressure. On the other hand, an increase in both dietary carbohydrate and blood cholesterol level reduced the risk of hypertension in subjects.

## 4. Discussion

The longer life expectancy and improper health behaviors can result a rise in the number of elderly who suffer from hypertension [14], especially in industrial areas [3]. Further studies are needed to identify the risk factors of hypertension and to know how to control it. Such research may prevent or delay the rate of disease and consequences in societies and in the elderly.

The current study evaluated the effects of socio-demographic factors, nutritional compounds, and blood components on the risk of hypertension in the Malaysian elderly. Based on the final model, female gender, and increase in the factors of body weight, dietary carbohydrate, and the blood levels of albumin, glucose, triglycerides, and cholesterol significantly influenced hypertension in respondents. Ethnicity prominently affected the risk of hypertension only at the first model. Females showed higher susceptibility than males to develop hypertension. The increase in factors, including body weight, and the blood levels of glucose, triglycerides, and albumin elevated the risk of hypertension in subjects as well.

Being overweight is a risk factor for a number of medical conditions such as hypertension [15,16]. Its effect on hypertension is likely through the renin-angiotensinogen level, sympathetic nerve activity, endothelial function [17], hypertriglyceridemia, and glucose intolerance [18]. Several pieces of evidence from epidemiological and clinical documents indicate a close relation among hypertension, dyslipidemia, and insulin resistance [19]. Triglycerides are the most common forms of fats in the body [20], and the metabolic disorder related to the abnormal concentration of triglycerides in the blood is hypertriglyceridemia [21]. The specific and direct role of hypertriglyceridemia in the pathogenesis of hypertension is not yet well defined [19].

However, triglycerides probably affect blood pressure through inducing endothelial dysfunction and the loss of endothelial vasomotor activity. Such changes may occur because of the initiation and enhancement of inflammatory responses of endothelial cells to cytokines, leukocyte recruitment [22], lipid oxidation [22], and the production of free radicals and endothelin-1. In addition, hypertriglyceridemia may enhance the risk of hypetnesion through sympathetic neural mechanism [19].

Hyperglycemia can increase the risk of hypertension [4] that is probably due to the concurrent presence of obesity, insulin resistance, and/or hyperinsulinemia [23]. Furthermore, hyperglycemia may itself lead to hypertension by changing vascular structure and function. Despite these findings, the long-term effect of hyperglycemia on hypertension is not yet well known [23,24]. In this study, the increased blood pressure following the increased blood level of albumin was also a confirmation to previous reports [25,26]. As albumin has a cardioprotective effect; therefore, this phenomenon is likely due to unrelated mechanisms [25] or the possible mediating effects of some factors such as tryptophan [26].

In this study, hypercholesterolemia reduced the risk of hypertension in the Malaysian elderly. It was in contrast to a previous report [27] indicating a positive correlation between the blood cholesterol level and high blood pressure. Such disparity is probably due to the influences of various factors, including smoking [28], education, body mass index [29], and the existence of metabolic diseases such as diabetes mellitus [30]. However, further studies are needed to identify the exact impacts of cholesterols on blood pressure.

As was found in the current study, high-dietary carbohydrate reduced blood pressure in the Malaysian elderly. It can be explained by the positive effect of high-dietary carbohydrate on the promotion of satiety, which can protect the body against being overweight, obesity, and, in turn, blood pressure [31]. However, further investigations are required to demonstrate the effects of carbohydrate intake on blood pressure. The result was consistent [32,33] and conflicting [34,35] with the research that studied the relation between high carbohydrate diet and blood pressure.

The results of the current study confirmed the previous reports [36,37], which mentioned that the risk of hypertension in older women is higher than older men. A possible reason is the lower level of hormones such as estrogen in the elderly women [38]. The longer life expectancy of women may enhance the rate of hypertension [39] probably because of age-related complications and further risk of facing stress, such as the loss of loved ones and even jobs [40]. At the same time, lifestyles, co-morbidities, and medications [40,41] may cause such disparity between genders as well. Contrary to several research reports documenting the positive effects of age [31,42] on the risk of hypertension, such correlation was not established in this study. In addition, despite the positive effect of sodium on hypertension in previous reports [43,44,45], such impact was not found in the current study. It can be explained by the state that hypertension occurs in people with sodium sensitivity, and, therefore, all people are not affected [46]. In addition, sodium may probably affect blood pressure via mediator influences. For example, the direct relation between sodium and medical conditions such as heart disease [47] and kidney dysfunction [48] may lead to high blood pressure in the elderly. Thus, factors affecting hypertension are probably different in older people and in various ethnics. Despite conflicting reports [49], this study found no relation between ethnicity and the risk of hypertension. It can be because of the effects of confounding factors and their interferences with ethnicity. However, ethnicity may sometimes impact health through cultural factors, migration, education, health beliefs, and socioeconomic status [50]. Genetic predisposing factors can also lead to differences in the prevalence of hypertension in populations [3]. However, many psychological, biological, behavioral, economic, and social circumstances affect the risk of hypertension, and, therefore, a broad knowledge about this literature is required in order to control disease.

## 5. Limitations of Study

The findings of the present study should be considered in light of its limitations, which can affect the interpretation of data about blood pressure in the elderly. One of the limits is the cross-sectional design of the study, which confines the determination of the exact effects of variables on the risk of hypertension. In addition, the physical and psychological co-morbidities in subjects can limit the appropriate assessment of the risk factors of hypertension. Diet and medications of subjects prior to the blood collection may also deviate the conclusion and restrict the study. In addition, the study is limited by the classification of variables using the median, which may cause a systemic bias and lead to some unexpected correlations, such as insignificant correlation between each variable of sodium and age with the risk of hypertension. However, conducting further research is required in order to identify the exact causes and risk factors of hypertension in the elderly.

## 6. Conclusions

It was concluded that female gender, enhanced body weight and increase in the blood levels of glucose, triglycerides, and albumin increased the risk of hypertension in subjects. Conversely, an increase in both blood cholesterol level and high-dietary carbohydrates decreased the risk of high blood pressure in respondents. Surprisingly, sodium intake did not have a significant effect on the risk of hypertension in the Malaysian elderly. However, further investigations are required to improve the knowledge about this issue, which enable a better understanding of risks and consequences in order to control hypertension.

## Figures and Tables

**Table 1:** Prevalence of hypertension among 2322 Malaysian elderly.

Character	*n*	*n* (%)	95% C.I.
Hypertension			
Yes	1059	45.61	43.59–47.64
No	1263	54.39	52.36–56.41

**Table 2:** Prevalence of hypertension and associations with socio-demographic factors.

	Whole	*n*	*n* %	95% CI	χ^2^	*p* Value
Age Groups						
<75 years	1848	846	45.78	43.52–48.06	0.11	0.742
≥75	474	213	44.94	40.52–49.44		
Gender						
Males	1114	477	42.82	39.94–45.75	6.71	0.010
Females	1208	582	48.18	45.37–51.00		
Marital status						
Married	1585	702	44.29	41.86–46.75	3.49	0.062
Non-Married	737	357	48.44	44.85–52.05		
Ethnicity						
Malays	1447	624	43.12	40.59–45.69	9.55	0.002
Non-Malays	875	435	49.71	46.41–53.02		
Education						
No	1830	846	46.23	43.96–48.52	1.35	0.246
Yes	492	213	43.29	38.98–47.7		
Body Weight						
≤60 kg	1160	457	39.4	36.63–42.24	36.49	<0.001
>60 kg	1154	599	51.91	49.03–54.78		

Significant at the 0.05 level using the chi-square test.

**Table 3:** Prevalence of hypertension and associations with nutritional factors.

	Whole	*n*	*n* %	95% CI	χ^2^	*p* Value
Sodium						
≤1500 mg	1401	670	47.82	45.21–50.44	6.10	0.14
>1500 mg	791	335	42.35	38.95–45.82		
Potassium						
≤1500 mg	1242	570	45.89	43.14–48.67	0.00	0.961
>1500 mg	950	435	45.79	42.65–48.97		
Calcium						
≤500 mg	1231	574	46.63	43.86–49.42	1.09	0.297
>500 mg	961	431	44.85	41.73–48.01		
Sugar						
≤18 mg	1095	496	45.3	42.37–48.26	0.27	0.604
>18 mg	1097	509	46.4	43.47–49.36		
Thiamin						
≤0.7 mg	1002	486	48.5	45.42–51.59	5.08	0.024
>0.7 mg	1188	519	43.69	40.89–46.53		
Energy						
≤1612 Kcal	1095	528	48.22	45.27–51.18	4.78	0.029
>1612 Kcal	1095	477	43.56	40.65–46.51		
Protein						
≤68 g	1061	501	47.22	44.23–50.23	1.51	0.219
>68 g	1130	504	44.6	41.72–47.51		
Carbohydrate						
≤212 g	1090	545	50.00	47.04–52.96	14.91	<0.001
>212 g	1101	460	41.78	38.9–44.72		
Fiber						
≤3.2 g	1034	461	44.58	41.58–47.62	1.26	0.262
>3.2 g	1158	544	46.98	44.12–49.86		
Cholesterol						
≤135 mg	1095	508	46.39	43.45–49.35	0.24	0.623
>135 mg	1096	497	45.35	42.42–48.31		
Fat						
≤50 g	1084	506	46.68	43.73–49.66	0.54	0.463
>50 g	1106	499	45.12	42.21–48.06		
Carbohydrate/Fat Ratio						
≤4.23	1092	521	47.71	44.76–50.68	1.86	0.173
>4.23	1098	492	44.81	41.89–47.76		

Significant at the 0.05 level using the chi-square test.

**Table 4:** Prevalence of hypertension and associations with blood factors.

	Whole	*n*	*n* %	95% CI	χ^2^	*p* Value
Glucose						
≤5.5 mmol	936	355	37.93	34.88–41.08	54.25	<0.001
>5.5 mmol	879	485	55.18	51.88–58.44		
Cholesterol						
≤5.3 mg	894	494	55.26	51.99–58.49	55.04	<0.001
>5.3 mg	888	335	37.73	34.60–40.96		
Triglycerides						
≤1.3 mg	916	401	43.78	40.60–47.01	6.03	0.014
>1.3 mg	861	427	49.59	46.26–52.92		
Albumin						
≤43 g	1113	479	43.04	40.16–45.97	14.00	<0.001
>43 g	669	349	52.17	48.38–55.93		

Significant at the 0.05 level using the chi-square test.

**Table 5:** Prevalence of hypertension and associations derived by logistic regression analysis.

	Model 1			Model 2			Model 3		
Variables	B	SE	OR	B	SE	OR	B	SE	OR
Gender	0.357 *	0.103	1.43	0.291 *	0.107	1.34	0.429 *	0.113	1.54
Ethnicity	−0.205 *	0.103	0.82	−0.203	0.107	0.82	−0.145	0.112	0.87
Body weight	0.629 *	0.103	1.88	0.642 *	0.103	1.90	0.477 *	0.109	1.61
Thiamin				−0.103	0.112	0.90	−0.139	0.117	0.87
Energy				0.142	0.149	1.15	0.168	0.155	1.18
Carbohydrate				−0.325 *	0.146	0.72	−0.305 *	0.152	0.74
Blood Glucose							0.654 *	0.106	1.92
Blood Cholesterol							−0.868 *	0.109	0.42
Blood Triglycerides							0.241 *	0.107	1.27
Blood Albumin							0.414 *	0.109	1.51

* *p* < 0.05; B = Coefficients; SE = Standard Error; OR = Odds Ratio.
